# Differential Effects of Bacitracin Methylene Disalicylate (BMD) on the Distal Colon and Cecal Microbiota of Young Broiler Chickens

**DOI:** 10.3389/fvets.2019.00114

**Published:** 2019-04-17

**Authors:** Alexandra Proctor, Gregory J. Phillips

**Affiliations:** Department of Veterinary Microbiology and Preventative Medicine, College of Veterinary Medicine, Iowa State University, Ames, IA, United States

**Keywords:** poultry, microbiota, antibiotic, 16S rRNA, growth promoting antibiotics

## Abstract

Antibiotics have been used extensively for growth promotion in poultry, along with other food production animals, as well as therapeutically to treat infectious diseases. However, with concerns over selection for drug antibiotic resistant bacteria the practice of using subtherapeutic doses of antibiotics is under increased scrutiny. Consequently, we assessed the impact of the commonly used antibiotic bacitracin methylene disalicylate (BMD) on the gastrointestinal microbiota of chickens. For this we administered therapeutic doses of BMD as a feed additive and 16s rRNA gene amplicon sequencing to measure changes in taxonomic abundance on the distal colon and cecal microbiota of young broiler chickens. While BMD treatment was found to impact the abundance of selected taxa and overall beta diversity, significant changes were, in general, limited to the colon of the treated birds. Selected taxa at the phylum, class, and genus levels that were most impacted were identified. The composition of the cecum remained relatively stable in BMD-treated animals. As poultry production practices seek alternatives to growth promoting antibiotic feed additives, manipulation of the gastrointestinal microbiota holds promise. These results suggest that targeting the cecum may offer a means to promote changes to the microbiota that maximize the benefits for the hosts.

## Introduction

Along with therapeutic use, antibiotics are well-established for their ability to promote growth through improved weight gain and feed efficiency in livestock (1), including in broiler chickens (2). In poultry, bacitracin methylene disalicylate (BMD) is commonly used for growth promotion (3). Compared to other growth promoting antibiotics, such as virginiamycin, BMD is a relatively narrow spectrum antibiotic that targets primarily gram-positive bacteria, including *Streptococci, Staphylococci, Clostridia, Fusobacterium*, and *Actinomyces*. BMD interferes with protein synthesis and cell wall production and induces cell lysis in these microorganisms (4, 5). The antibiotic is not well-absorbed by the intestine and therefore primarily acts on bacteria in the gastrointestinal (GI) tract of the animals through delivery as a feed additive (6).

Feeding chickens low doses of BMD benefited the birds, including increased villus height throughout the small intestine and improved digestion of dietary components that correlated with increased body weight and feed consumption (7).

BMD is also used as to treat and prevent necrotic enteritis caused by *Clostridium perfringens* (8, 9), which is a cause of significant economic loss in the poultry industry (10). While antibiotic growth promoters make important contributions to the overall efficiency of livestock production, they are also not without their concern as sub-therapeutic doses used are also associated with selection and spread of drug resistant bacterial pathogens (11, 12). Concerns over widespread use of antibiotics in agriculture has prompted a ban on their use in the European Union with increased scrutiny for their use in the United States (13).

Given the importance of poultry for human nutrition and the food animal industry world-wide, emphasis has been placed on characterizing the chicken microbiome as a means to improve our understanding of antibiotic growth promotion and to identify alternative strategies that do not select for drug resistant bacteria (14–18). Toward this, numerous studies, representing a variety of methods, have assessed the impact of antibiotic treatment on the microbiota of poultry (7, 9, 19–32). In general, these studies have shown that growth-promoting antibiotics can have significant effects on the structure and function of the microbiota colonizing the GI tract. As to be expected with a list of wide-ranging studies, there are few bacterial taxa that are consistently altered by antibiotics that can explain their growth promoting activities since the composition and activity of the chicken microbiome is highly dependent on environmental conditions, feed composition and method of assessment of the microbioal communities. Interestingly, however, chicken microbiota studies have revealed that BMD, along with other growth-promoting antibiotics, can deplete species of *Lactobacillus*, as well as other probiotic species (32, 33). This observation has led to the suggestion that a reduction in bile-salt hydrolase activity encoded by many of these bacteria may contribute to growth promotion by reversing the negative effects on fat metabolism of these enzymes (34). Clearly additional studies are needed to better understand how changes to the microbiota by low-dose antibiotics contribute to animal growth enhancement.

To further out understanding of how the growth promoting feed additive BMD impacts the chicken microbiota, we have focused on distinguishing between the effects of the antibiotic on cecal vs. colon bacterial populations. These two compartments of the chicken digestive tract are colonized with distinct microbial communities (35, 36). Also, while metabolism and adsorption of macronutrients occurs primarily in the colon, fermentation of complex polysaccharides occurs primarily in the cecum (14, 15, 36). Because of these spatial and functional differences, we sought to determine the extent to which BMD impact the microbiota of the distinct compartments of the GI tract. For this, we conducted 16s rRNA gene amplicon taxonomic profiling of the microbiota of the distal colon and cecum from young broiler birds using therapeutic doses (8, 37) of the antibiotic to accentuate differences in microbial composition in the GI tract.

## Materials and Methods

### Animal Model and Housing

This study was carried out with the approval of the Iowa State University Institutional Animal Care and Usage Committee under protocol number 6-11-7167-G. The design followed a necrotic enteritis model, however, no pathogens were administered to the chickens and only antimicrobial feed additives were added to the experimental group.

Approximately 30 day old jumbo Cornish/Rock broiler chicks were obtained from Welp Hatchery (Bancroft, IA) and housed in pens created by tying two 32″ × 8′ × 1/8″ (81 cm × 2.45 m × 0.3 cm) pegboards together to form a circle. This circular pen was divided into three equally sized areas with similar pegboard material. Each pen was bedded using ~3″ (7.5 cm) of wood shavings. Heat lamps were made available for each pen. One two-gallon, galvanized waterer and one galvanized metal feeder were supplied to each pen.

Groups of 15 birds were housed in the pens described. On days 1–7, each group received 1 kg of a low-protein chick starter (LPF) once a day. On days 8–10, each group was given 1 kg of a high protein feed (HPF) once a day. On days 11–18, the control group remained on the same HPF feed while the challenge group received the HPF supplemented with BMD (200 g/ton). On these days, each group was fed 1 kg HPF with or without BMD twice a day. On day 19, the chicks were euthanized and samples were collected. Distal colon and cecal contents were collected and stored at −80°C until total DNA was isolated.

### DNA Isolation

Total genomic DNA was extracted using the PowerSoil DNA Isolation Kit (MoBio, Carlsbad, CA). The manufacturer's protocol was followed with the exception of the initial vortexing step, which was extended to 20 min to thoroughly homogenize the samples. Purified genomic DNA extracts were quantified using a Quibt 2.0 Fluorometer (Life Technologies, Carlsbad, CA), and DNA stored at −20°C in the provided 10 mM Tris buffer.

### Sequencing and Analysis

PCR amplification of the variable regions 3–5 of the 16S rRNA gene was done using region specific primers 357F (CTCCTACGGGAGGCAGCAG)−926R (CCGTCAATTCMTTTRAGTTT). Amplicon sequencing was performed at the Research and Testing Laboratory (Lubbock, TX) using the Roche 454 Titanium platform.

The resulting DNA sequences were analyzed using QIIME (Quantitative Insights into Microbial Ecology) (38). The reads were first demultiplexed and binned per sample. The reads were also quality filtered during this step to remove poor quality sequences using default quality filtering values. Denoising of the 454 reads was performed using Denoiser (39). Chimeric sequences were identified using USEARCH and removed (40, 41). The remaining sequences were clustered into OTUs at 97% similarity using USEARCH and the open reference OTU picking strategy in QIIME. Sequences were aligned to the Greengenes (13_5) rRNA sequence core reference database using PyNAST (42, 43). Taxonomic assignments were made using the RDP Classifier 2.2 at a 97% similarity to the Greengenes reference database (44). Phylogenetic trees were built using FastTree 2.1.3 (45). Alpha and beta diversity, analysis of similarity (ANOSIM) and Adonis tests were generated using QIIME. PCoA plots were generated using Emperor in QIIME (46). Mann–Whitney *U*-tests were performed on taxonomic summaries using a custom R script (R Project) developed at the Institute of Genome Sciences at the University of Maryland-Baltimore. Sequence reads have been submitted to NCBI's short read archives.

## Results

### Alpha and Beta Diversity

A total of 79,670 sequences were analyzed using QIIME. After filtering based on quality scores, 73,529 sequences corresponded to 619 OTUs with an average of 2,298 ± 1,335 sequences per sample.

**Figure 1:**
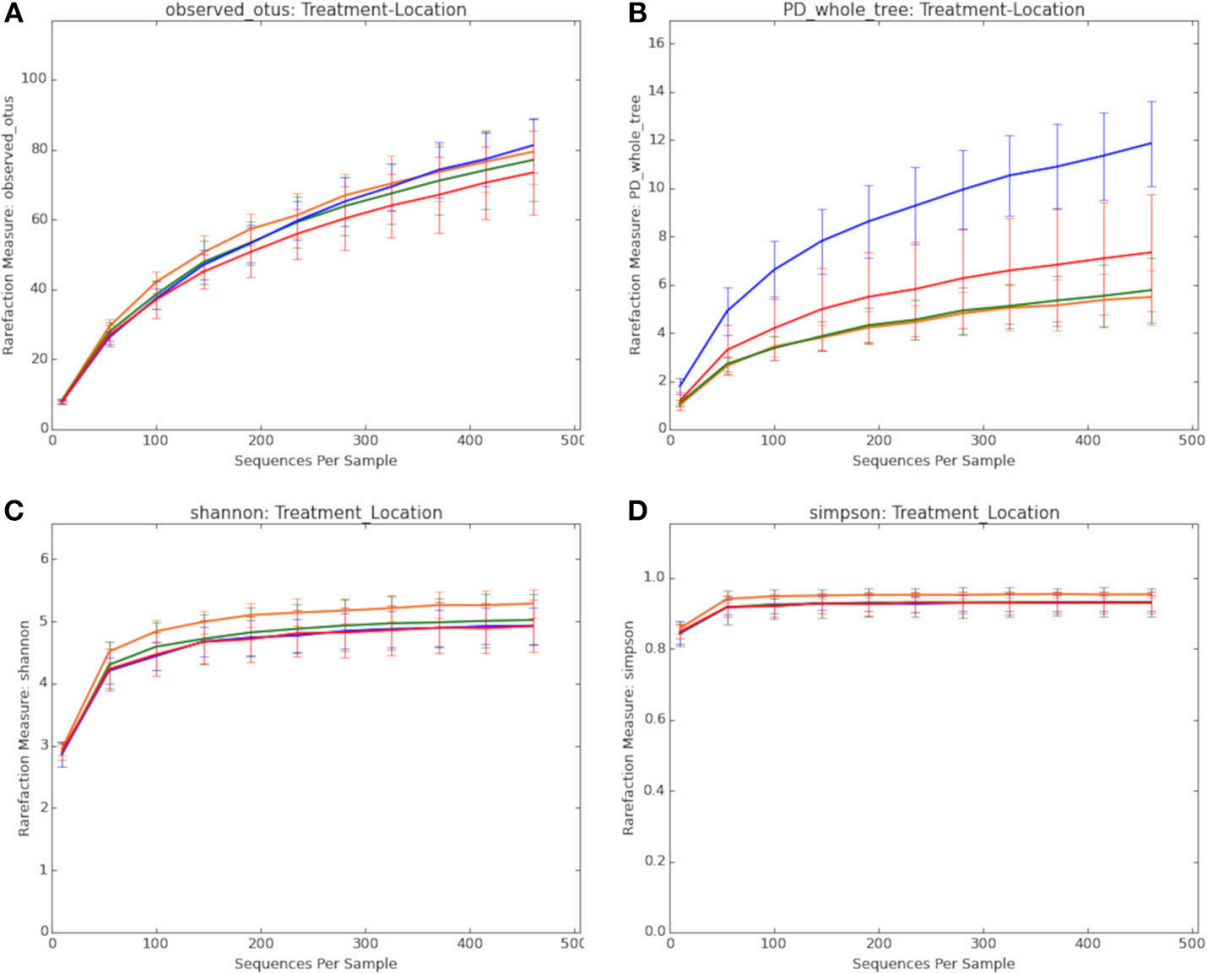
Comparison of the composition of the microbiota in treatment groups. **(A)** Observed OTUs. **(B)** Faith's phylogenetic diversity. **(C)** Shannon alpha diversity index. **(D)** Simpson alpha diversity index. Treatment groups include: BMD treated, cecum (red line); BMD treated, distal colon (blue line); Control, cecum (orange line); Control, distal colon (green line).

Figure 1 summarizes the alpha diversity measurements used to determine the extent to which BMD altered the composition of the microbiota. This included measurements of observed OTUs (1A), Faith's phylogenetic diversity (1B), Shannon (1C), and Simpson (1D) alpha diversity indices to compare treatment groups. Non-parametric two-sample *t*-tests were used to identify significant differences among the groups. While significant differences in OTUs were not observed among the groups by the Simpson and Shannon diversity metrics (data not shown), differences were noted by Faith's phylogenetic diversity (Table 1).

**Table 1:** Significant differences among the treatment and control groups as assessed by Faith's phylogenetic diversity non-parametric two-sample *t*-tests.

**Group 1**	**Group 2**	**Group 1 mean**	**Group 1 std**	**Group 2 mean**	**Group 2 std**	***t* statistic**	***p*-value**
Control-cecum	BMD-cecum	5.48	1.13	7.32	2.41	−1.80	0.66
BMD-cecum	BMD-colon	7.33	2.41	11.86	1.76	−3.89	0.04
Control-cecum	Control-colon	5.48	1.13	5.77	1.32	−0.43	1
BMD-colon	Control-colon	11.86	1.76	5.76	1.32	7.30	0.01

Within the BMD treated group, the distal colon showed higher phylogenetic diversity compared to the cecal samples (*p* = 0.036). The phylogenetic diversity of the colon of the BMD treated birds was also greater than the same samples from the control group (*p* = 0.006). This could indicate the BMD treatment is causing a few OTUs to become depleted hence allowing for other unique OTUs to establish or increase in their proportion within the community.

**Figure 2:**
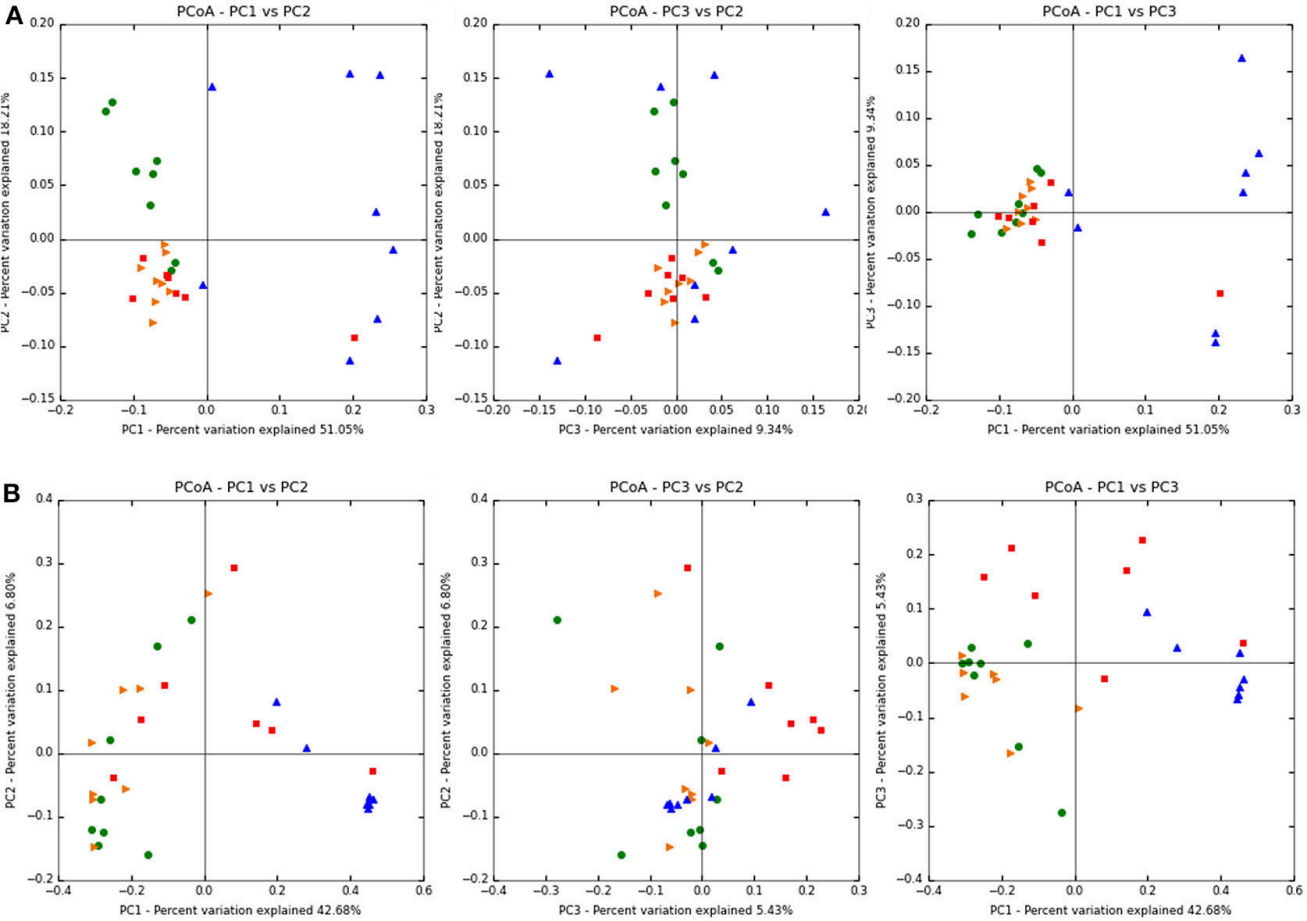
Unifrac PcoA plots of the treatment groups. **(A)** Weighted **(B)** unweighted. Treatment groups include: BMD treated, cecum (red squares); BMD treated, distal colon (blue triangles); Control, cecum (orange triangles); Control, distal colon (green circles).

Figure 2 shows the weighted (abundance considered) Unifrac PcoA beta diversity plots of the treatment groups (Figure 2A), while Figure 2B shows the unweighted (abundance independent) plots. In the weighted Unifrac PCoA analysis, cecal samples from both treated and control groups showed greater similarity than samples from the colon. Conversely, the colon samples of the control and BMD groups showed greater variability, with less distinct clustering. Also, the control samples from the colon clustered closer to each other and also closer to the cecal samples than the BMD colon samples.

The unweighted Unifrac PCoA plots showed that treatment influenced the dissimilarity of the samples more than GI tract location as samples in each group were not clustered as tightly as the weighted PCoA. Analysis of similarity (ANOSIM) and Adonis tests were performed on the weighted and unweighted Unifrac distances obtained from the beta diversity workflow in QIIME. The ANOSIM test based on both treatment and GI location resulted in a *p*-value of 0.001 and a test statistic of 0.379 for weighted and a *p*-value of 0.001 and test statistic of 0.556 for unweighted Unifrac analysis. These metrics indicated that the grouping based on the variables of treatment and GI location is weak (i.e., the differences can be explained by randomness). Adonis tests also based on both treatment and GI location resulted in a *p*-value of 0.001 and an *R*^2^ value of 0.441 for weighted and a *p*-value of 0.001 and *R*^2^ value of 0.404 for unweigthed Unifrac analysis.

### Relative Abundance

**Figure 3:**
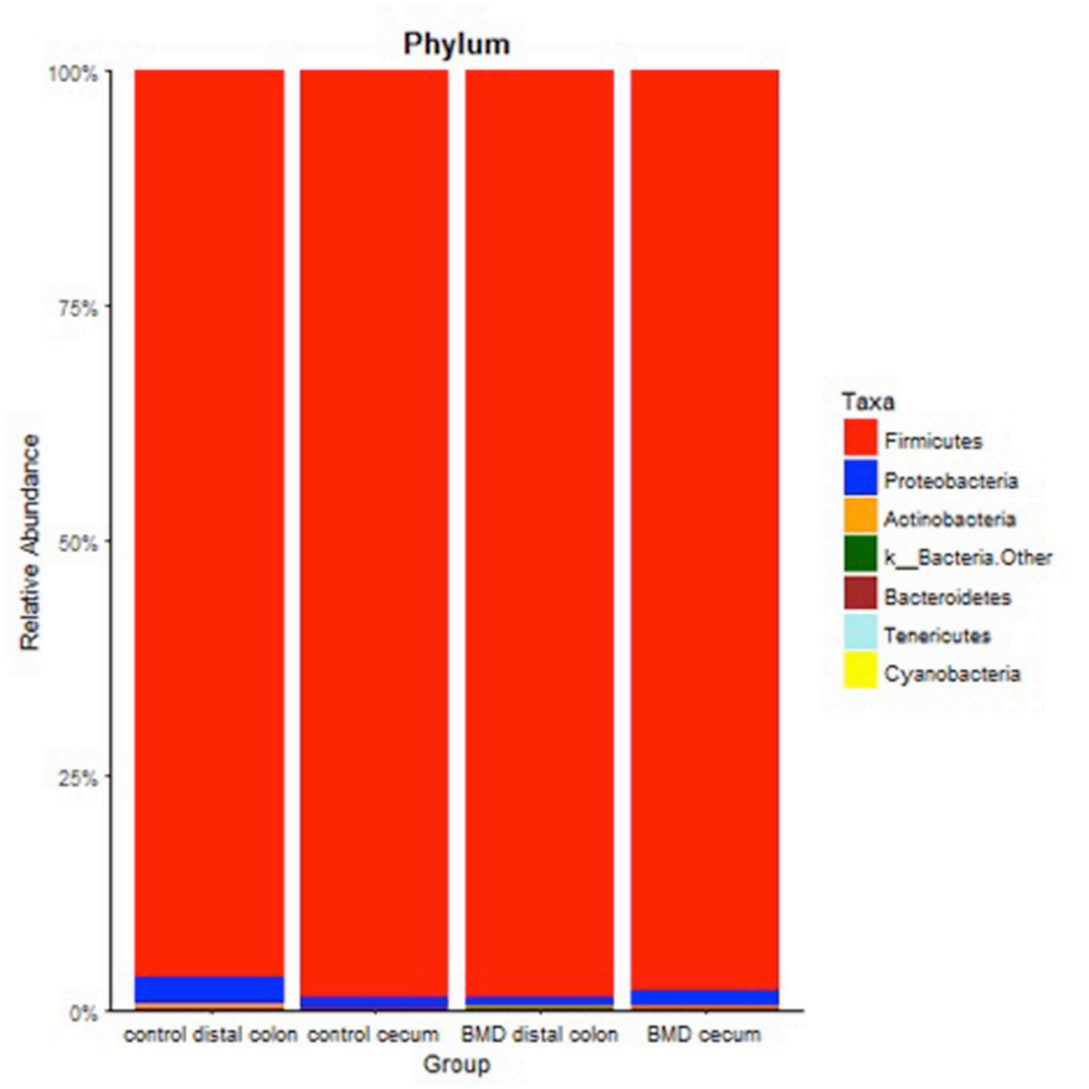
Relative abundance at the phylum level for each of the treatment groups. Specific phyla are shown in the key on the right.

Differences in relative abundance among all the treatment groups at different taxonomic levels were assessed using Mann–Whitney U-tests. Figure 3 shows the taxonomic summary for each group at the phylum level and Table 2 shows the *p*-values for the Mann–Whitney U-tests for phylum level differences. As evident, the dominant phylum for each treatment group in both the distal colon and cecum was *Firmicutes* (96.5–98.7%), with other phyla including *Proteobacteria* (0.9–3.0%), *Actinobacteria* (0.1–0.5%), and *Bacteroidetes* (~0.1%).

**Table 2:** Bacterial taxa with significant differences in abundance in pairwise comparisons between treatment groups as determined by Mann–Whitney *U*-tests.

**Taxonomic rank**	**Treatment Groups**^**a**^				**Taxa with significant differences**	**Mann–Whitney *p* value**
	**Control**		**BMD**			
	**Co**	**Ce**	**Co**	**Ce**		
Phylum					*Proteobacteria*	0.043
Class					*Bacilli*	0.019
Class					*Clostridia*	0.007
Genus					*Lachnospiraceae*	0.008
Genus					*Oscillospira*	0.008
Genus					*Erysipelotrichaceae* cc_115	0.034
Genus					*Enterococcus*	0.009
Genus					*Peptostreptococcaceae*	0.035
Genus					*Lachnospiraceae*	0.049

a *Co, colon; Ce, cecum. Filled cells show pairwise comparisons associated with significant differences in taxonomic abundance*.

**Figure 4:**
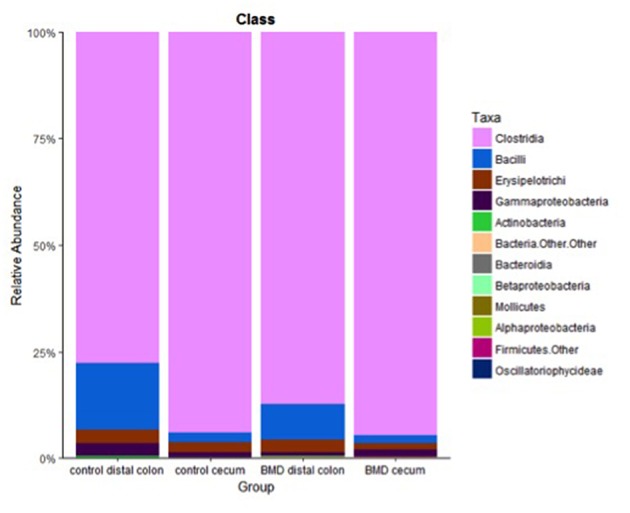
Relative abundance at the class level for each of the treatment groups. Specific classes are shown in the key on the right.

At the class level (Table 2), *Bacilli* were depleted in the cecum of the control fed group compared to the distal colon site (*p* = 0.01865) and *Clostridia* were enriched in the cecum of the control birds compared to the distal colon (*p* = 0.007). Figure 4 shows the relative abundance of the classes from each treatment group. The *Firmicutes Clostridia* and *Bacilli* were the dominant class (77.7–94.5 and 2.0–15.6%, respectively), with the remaining classes including *Erysipelotrichi* (1.5–3.2%), *Gammaproteobacteria* (0.9–2.9%), and *Actinobacteria* (0.1–0.5%). The *Clostridia* appeared to comprise a greater relative abundance in the cecum (84.3–94.5%) of both treatment groups compared to the distal colon (60.2–87.2%). There were no significant differences between the cecal samples of the control and BMD treated groups or the cecal samples and the distal colon samples of the BMD group.

**Figure 5:**
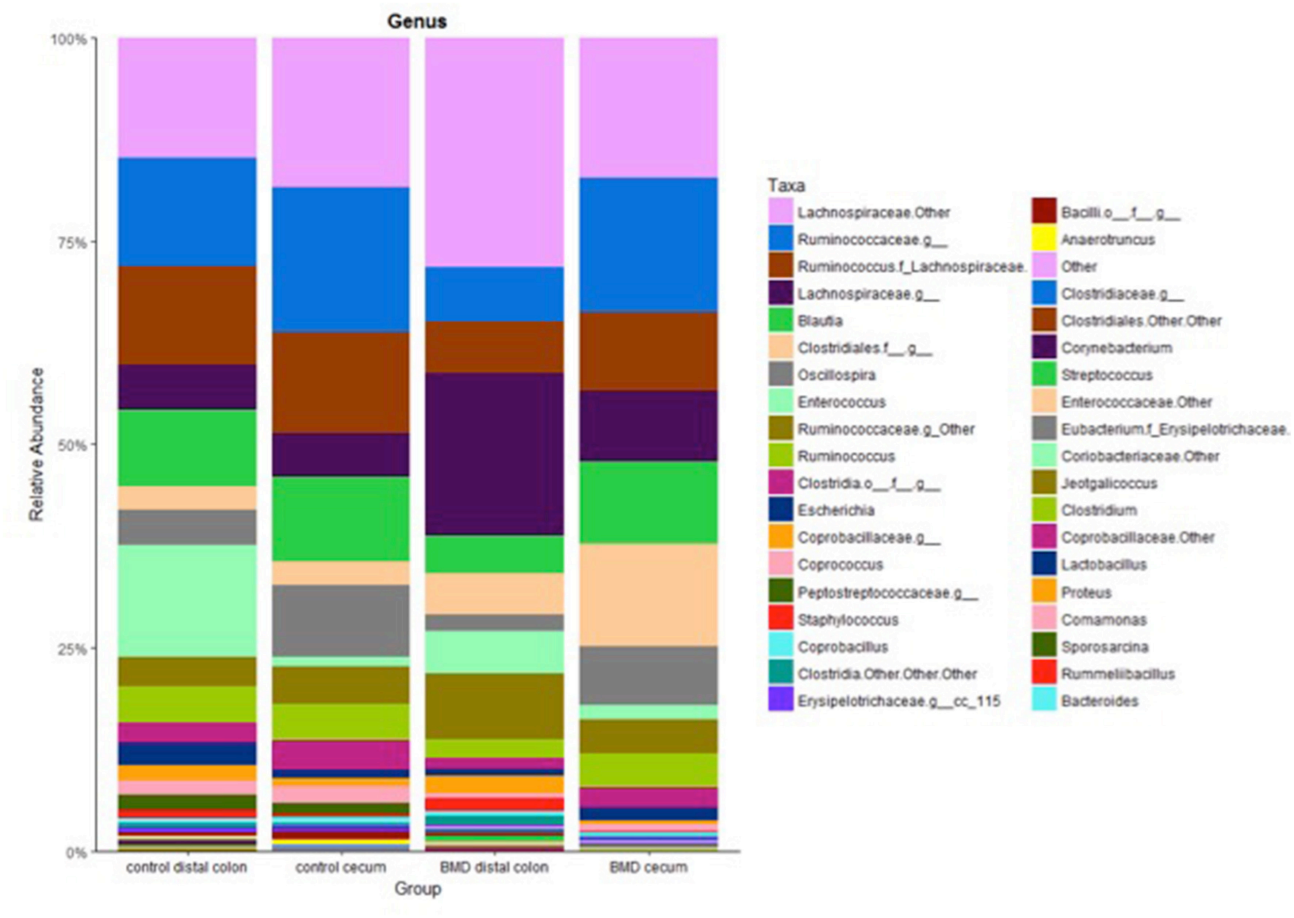
Relative abundance at the genus level for each of the treatment groups. Specific genera are shown in the key on the right.

Selected genera were also significantly altered by the BMD treatment (Table 2). Specifically, two genera, *Oscillospira* and an unnamed member within the *Erysipelotrichaceae* family (cc_115), were depleted in the distal colon of the BMD supplemented group compared to the distal colon of the control group (*p* = 0.008 and 0.034, respectively). Conversely, an unknown genus in the family *Lachnospiraceae* was enriched in the distal colon of the BMD treated group compared to the distal colon of the control group (*p* = 0.008). The same microorganism was also depleted in the cecum compared to the colon of the BMD treated birds (*p* = 0.049). Only an unknown genus in the family *Peptostreptococcaceae* was depleted in the cecum of the BMD treated group compared to the cecum of the control group (*p* = 0.034). Figure 5 summarizes the genera that comprised each of the treatment groups and reveals that no one genus dominated in abundance.

## Discussion

Consistent with previous studies, the distal GI tracts of the birds surveyed here were dominated by *Firmicutes* (Figure 3), which included the classes *Clostridia* and *Bacilli*. Members of the phyla *Proteobacteria, Actinobacteria, Bacteroidetes*, and *Tenericutes* were also detected (47–49). The differences in the distribution of the microbiota were more evident at the class and genus level with the greatest changes observed in the distal colon in the BMD treated birds. Faith's phylogenetic diversity revealed an increase in diversity in the colon of the BMD treated birds. One explanation for this is that the antibiotic treatment depleted some gram-positive species that allowed others to expand in their place without significantly altering the total number of OTUs observed within the samples. In general, the composition of cecal samples showed more similarity than samples recovered from the distal colon for both the control and BMD treated birds. There were also fewer changes between the cecal samples of the control and BMD groups compared to the distal colon samples. The observed differences in the microbial populations and relative stability of the cecum is consistent with previous studies comparing cecal populations and feces in broiler chickens (36), and indicates that the cecal microbiota is buffered to some extent from the antimicrobial effects of BMD. This likely holds true for other feed additives in chickens (31, 50).

Individual variation was more also evident in the distal colon samples than the cecal samples. Bird to bird variation is not uncommon in chickens and may be explained by the immediate environment having significant impact on how domestic fowl acquire their microbiota over maternal sources as is observed in most mammals. Chicks hatched in commercial settings are typically not exposed to the hen's microbiota post hatching, therefore colonization depends on environmental factors and could be affected by the surroundings, litter management practices, and contact with other chicks (50, 51).

As cited in the introduction, there have been several studies showing that sub-therapeutic doses of antibiotics can alter the microbiota. To enhance these effects, we used a BMD dose considered to be therapeutic designed to reduce potential pathogens during outbreaks of GI diseases such as necrotic enteritis (8, 37). At this dosage, the Phylum *Proteobacteria* was reduced (*p* = 0.04) in the distal colon of the BMD treated birds when compared to the control. BMD treatment also decreased *Oscillospira, Peptostreptococcaceae*, and an unknown *Erysipelotrichaceae* in the colon of the birds, while only *Peptostreptococcaceae* was depleted in the cecum of the BMD group.

Between treatment groups, the control samples clustered closer together in the Unifrac PCoA plots compared to the BMD groups. The individual variation among birds, as well as variability in feed consummation may have contributed to the lack of tight clustering in the BMD groups. “Pecking order” among the birds may also influence feed (and BMD) consumption within the groups (52).

In general, few OTUs were significantly different between the control and BMD treated group in the distal colon. While the more proximal GI has greater susceptibility to antibiotics than the distal GI, this may also indicate the bacteria in the distal colon of the chicks have a higher proportion of bacteria that are resistant to bacitracin (22, 53, 54).

These results have implications for development of new strategies as use of antibiotics for growth promotion is being phased out of commercial use. Specifically, selective manipulation of the microbiome through alternative supplementation is growing in interest as an alternative to antibiotics (55, 56). This can include the use of beneficial bacteria as probiotics, prebiotic supplementation, phytobiotics, or enzymes (30). Antibiotic alternatives can confer resistance to colonization of pathogens through competitive inhibition, decreasing pH of the GI tract, or by contributing to overall animal health through immune modulation (55, 57, 58).

Probiotics can consist of one or more Gram-positive bacteria, such as *Lactobacillus, Enterococcus*, or *Bacillus*, as well as multiple strains of the same species. For example, Nisbet found administration of 29 microorganisms from ceca of older, *Salmonella* free chicks protected 1-day old chicks from various enteric pathogens (59, 60). Probiotics also have potential to replace growth-promoting antibiotics as evidenced by studies showing broilers fed *Bacillus subtilis* daily had increased weight gain and improved feed conversion ratios than the control animals (58). Additional benefits of probiotics include enhanced production of short chained fatty acids (butyrate, acetate, and proprionate) in broilers fed non-digestible carbohydrates or oligosaccharides that are fermented by members of the microbiota, as well as enhanced protection and antimicrobial production (30). Prebiotics can increase adaptive immune responses when administered *in ovo* and aided in intestinal development in newly hatched chicks (61).

The relative stability of the chicken microbial community in the cecum compared to the distal colon may prove to be beneficial to production practices that seek to exploit the microbiome to enhance production. For example, modern poultry production typically prevents contact between chicks and older birds. This means the chicks are exposed to environmental bacteria rather than those associated with a healthy bird. Cecal transplants, or beneficial bacteria sourced from the cecum, could be used as “seeds” for post hatched chicks. While more studies are needed, the cecal microbiota presents itself as a potential source for colonizing newly hatched chicks with a healthy, chicken specific community that can speed GI development and help prevent diseases such as necrotic enteritis.

## Ethics Statement

This study was carried out with the approval of the Iowa State University Institutional Animal Care and Usage Committee under protocol number 6-11-7167-G.

## Author Contributions

AP designed and conducted the experiments, analyzed the data, and wrote the manuscript. GP assisted with data analysis and helped write the manuscript.

### Conflict of Interest Statement

The authors declare that the research was conducted in the absence of any commercial or financial relationships that could be construed as a potential conflict of interest.
